# Feasibility and Utility of Multimodal Micro Ecological Momentary Assessment on a Smartwatch

**DOI:** 10.1145/3706598.3714086

**Published:** 2025-04-25

**Authors:** Ha Le, Veronika Potter, Rithika Lakshminarayanan, Varun Mishra, Stephen Intille

**Affiliations:** Northeastern University, Boston, Massachusetts, USA; Khoury College of Computer Science, Northeastern University, Boston, Massachusetts, USA; Khoury College of Computer Science, Northeastern University, Boston, Massachusetts, USA; Northeastern University, Boston, Massachusetts, USA; Khoury College of Computer Sciences and Bouve College of Health Sciences, Northeastern University, Boston, Massachusetts, USA

**Keywords:** Ecological momentary assessment, Experience sampling, Ubiquitous computing, Wearable computing, Speech input, Touch input, Multimodal input

## Abstract

*μ*EMAs allow participants to answer a short survey quickly with a tap on a smartwatch screen or a brief speech input. The short interaction time and low cognitive burden enable researchers to collect self-reports at high frequency (once every 5–15 minutes) while maintaining participant engagement. Systems with single input modality, however, may carry different contextual biases that could affect compliance. We combined two input modalities to create a multimodal-*μ*EMA system, allowing participants to choose between speech or touch input to self-report. To investigate system usability, we conducted a seven-day field study where we asked 20 participants to label their posture and/or physical activity once every five minutes throughout their waking day. Despite the intense prompting interval, participants responded to 72.4% of the prompts. We found participants gravitated towards different modalities based on personal preferences and contextual states, highlighting the need to consider these factors when designing context-aware multimodal *μ*EMA systems.

## 1 Introduction

Accurately detecting human behaviors is an important research area in ubiquitous computing, human-computer interaction, and personal health informatics. Researchers can use behavior recognition models to drive context-aware interactive systems, just-in-time interventions, or health monitoring tools. Building behavior inference systems requires datasets that have *high-fidelity labels of behavior* that can be used to effectively train models. Systems that can be used to collect *temporally-dense*, in-situ behavioral data may result in datasets that could be used to help build and validate behavioral recognition models. Such labeled data might also be used to create personalized recognition models that may accurately detect behavioral patterns.

A particularly important human activity recognition (HAR) task in health and other domains is the detection of human posture, physical activity (PA), and sedentary behaviors from wearable sensors. Effectively training, validating, and benchmarking such HAR models, however, requires large, continuously labeled datasets. Most wearable HAR datasets are collected in heavily controlled [36] or semi-controlled [18] settings; the resulting datasets may not reflect the diversity and complexity of activities that people engage in during daily living. Models trained on such datasets, therefore, often perform poorly when tested on data from real-world, less-controlled scenarios [112] – where people have a wider range of activities or move their bodies more naturally. For example, when someone is told to *sit* in the lab, they may move in a controlled way and sit upright, but when they *sit* in real-life, they may plop down on a couch and lounge. Moreover, some activities, such as *driving*, are common in everyday life but difficult to capture realistically in lab protocols. The gold-standard method used to collect labels in-the-wild is to use an egocentric on-body camera to record images or video; the visuals are then used to label posture and behaviors post-hoc [14, 32]. For researchers, labeling the images or videos [45] is time-consuming, tedious and resource-intensive. Further, because human labeling is involved, the method cannot be used for developing systems that gather new labels in real-time and update models in-situ. For the participants in studies, the burden of wearing the front-facing, on-body camera can raise significant privacy concerns [46] about recording the activities of the participant *and* those nearby; thus participant recruiting can be challenging.

Ecological Momentary Assessment (EMA) is a data collection method whereby participants in research studies are prompted to complete short surveys periodically, often on their smartphone [98]. *μ*EMA is a specific form of EMA where where researchers prompt participants to answer a single multiple-choice question using an “at-a-glance,” single-tap interaction, often on a smartwatch screen [43]. Researchers have extended *μ*EMA to enable participants to provide their behavioral labels using speech input, triggered by a vibration on a smartwatch or a *beep* heard through an earable [59, 91]. By design, each *μ*EMA prompt delivers only a single question that can be answered quickly, which enables *μ*EMAs to be prompted at a high interval (e.g., once every 5–15 min) while maintaining a high response rate and thus ensuring temporally dense labels. One challenge with deploying *μ*EMAs is that there may be contextual biases in response rates that depend, in part, on the modality of data entry (e.g., participants may be less inclined to speak to the watch in public settings or tap on the watch during vigorous exercise) [59, 83].

In this work, we combine speech and touch input to allow **multimodal** *μ***EMA**, giving participants maximum flexibility when self-reporting behaviors (in this case their posture and activity). We explore the impact of multimodal interaction on reporting burden, where our aim is to test a methodology that may allow participants to maintain a high compliance rate despite data collection using a temporally intensive prompting interval—once every five minutes. Our research questions are:

- **RQ1:** What is the usability and feasibility of using multimodal *μ*EMA to collect temporally dense posture and physical activity labels in-the-wild?
- **RQ2:** What factors affect participants’ non-response and modality choice when responding to multimodal *μ*EMA prompts?
- **RQ3:** What are the characteristics and potential utility of the posture and physical activity labels collected with multimodal *μ*EMA?

To address these questions, we conducted a seven-day field study with 20 participants. The key contributions of our work are:

- We introduce a new multimodal *μ*EMA data collection method that allows speech and/or touch input. We explore the usability of our system in the context of collection of posture and activity labels by conducting a mixed-method field study with 20 participants for seven days.
- We quantitatively show that on our acquired dataset, passively sensed contextual parameters (heart rate, wrist movements, location, ambient noises, phone usage, time of day and day of week) are associated with response rate and choice of interaction modality. Furthermore, we qualitatively examine how interruption and interaction burden are associated with multimodal *μ*EMA.
- We explore the characteristics and utility of labels collected using our system, and we demonstrate that automatic, real-time label extraction is possible using an adapted commercial speech recognition model and an open-source large-language model.

## 2 Related Works

This work builds on prior research on ecological momentary assessment (EMA), multimodal input, and in-situ data collection – the methods that have been used to collect human behavioral data.

### 2.1 Collecting in-situ behavioral labels using ecological momentary assessment (EMA)

Ecological momentary assessment [95], sometimes called the experience sampling method, is a data collection method widely used in behavioral monitoring research to collect longitudinal data [19, 98]. Using EMA, researchers can collect ecologically valid measurements by using notifications on a mobile phone or wearable device to prompt participants to report data in-situ. The primary disadvantage of EMA is that the participants may perceive notifications as burdensome *because* they are prompted in-situ; pulling out a device and stopping an ongoing activity to answer surveys can disrupt the behaviors being measured. Thus, most research studies seek to balance compensation and burden to ensure a high response rate to EMA [111]. *μ*EMA [43, 52] is a modified version of the standard EMA method where each prompt is guaranteed to include only a single-question survey, often presented on the smartwatch, that can be answered with a quick tap; *μ*EMAs (microinteraction EMAs) are designed to be answerable with “at a glance,” single-tap interactions that take only 2–3 s. Prior research has demonstrated that even when *μ*EMA surveys are delivered at rates of up to four times an hour, participants in research studies can maintain a response rate significantly higher than for standard EMA [43, 82]. *μ*EMAs can be implemented well on a smartwatch, because the watch is easily accessible on the wrist. One limitation of *μ*EMA delivered on a smartwatch, however, is the limited amount of space available on the watch screen, which reasonably can only support multiple-choice questions with less than five options. While this limitation helps ensure *μ*EMA questions do not become too complex, it also makes *μ*EMA more suitable as a prediction confirmation mechanism (e.g., “Are you walking?” “Yes/No”) than for input that involves selecting from a list of possibilities (e.g., "What are you doing now?" with a long list of activities). Another consideration when using smartwatch-based *μ*EMA is that answering a question requires a two-handed interaction, which can be inconvenient during certain activities (e.g., driving or carrying groceries). Audio-*μ*EMA [59] allows participants to use speech input to provide open-ended responses. In audio-*μ*EMA, the system prompts participants using either a short acoustic cue presented through an earable, or if a watch is used, through a vibration on the wrist; the prompt indicates that the participant should speak the answer to a known question (e.g., what is their in-the-moment behavior). Due to the hands-free nature of speech interaction [89], audio-*μ*EMA could allow capture of a wide range of behavioral labels (e.g., postures, activities, or contextual information) while maintaining a high response rate.

A limitation of all EMA-based implementations is that in-the-moment contextual parameters may affect response rate [83]; this contextual reactivity could impact the validity of some types of behavior data collected using this method (e.g., if participants are unwilling to respond while exercising, we would not gather any labels on this activity to train recognition models). Thus, combining different modalities of EMA and a retrospective recall may be required to capture a comprehensive picture of a participant’s entire waking day. Multimodal EMA systems have been developed for home environments [63, 110]; in that work, even though participants preferred touch interactions to voice commands, modality preferences varied based on participants’ contextual states. However, we are aware of no research to date on using a multimodal *μ*EMA system in free-living settings.

### 2.2 Multimodal data logging and tracking systems

People interact with the world around them by speaking, touching, gesturing, drawing, and pointing; they use different modes, alternatively or simultaneously, in different contexts [94]. Multimodal input interfaces can improve user experience and system robustness [78] on multiple tasks such as data analytics/exploration [49, 51, 92, 101], tracking exercise, [66] tracking food intake [53, 65, 67, 96], and in-car communication [44, 54, 58, 104].

Touch is the most common input modality used in health-tracking applications, mainly due to the ubiquitous nature of commercial smartphones and smartwatches. Recently, there has been an increase in research on voice-based interfaces for behavioral logging [4, 51, 66]. Researchers have implemented voice-based interfaces on many device form factors because voice does not require a physical interface. Although researchers reported users’ positive reactions to speech input, the method presents challenges related to addressing cognitive load, social context, and speech recognition errors [96]. Other modalities like touch or keyboard often complement speech inputs to allow error reconstruction [78]. Researchers have also explored the uses and combination of different input modalities (e.g., voice log [38, 65, 67, 96], photos [27, 28, 65, 69], touch [65, 96], and type [69]) on different device form factors (e.g., mobile [65, 67, 69, 96], desktop [69], smartwatch [4, 38, 50], and smart speaker [63]) for behavioral journaling/tracking.

Even if a system allows multimodal interaction, users are not guaranteed to interact multimodally [77]. A user’s modality usage patterns are heavily influenced by external and task contexts. Researchers have shown modality usage can be affected by the cognitive and communication load of the task [79], contextual variables (e.g., surrounding environments, movements/activities, hand usage, visual load) [61, 87], and physical/mental interaction effort [13]. In this paper, we investigate the usability of our multimodal *μ*EMA system and examine different contextual variables that affect user modality choice, given the task of recording posture and physical activity labels.

### 2.3 Collecting human activity labels in-the-wild

Capturing information about a person’s free-living physical activities (PA) labels in-the-wild can support better training of HAR models and more realistic evaluation of such models. One approach is to use participants’ self-reported data, often acquired either via end-of-day survey [8], in-situ measurements (EMA) [85], or a mixture of both [41, 106]. Labels collected using end-of-day recall surveys tend to suffer from recall bias, in which events happening before or after can affect the recalled event. Fast-changing, overlapping sequences of activities [6] can often lead to mistakes in labeling activity boundaries, which can significantly reduce model performance [55]. An alternative approach to self-report is to use a body-worn camera, where participants wear a front-facing camera around their neck [24, 34, 47] or on their head [32] to capture an egocentric narrative of their daily life. Although this approach does not create a response burden for the participant, it introduces privacy concerns that may hinder participant recruitment and is not sustainable for longitudinal studies. Furthermore, labeling a large volume of video data after-the-fact is time-consuming. The quality of annotating videos retrospectively relies on the annotators’ ability to extract information about a person’s activity from the first-person narrative video without any additional self-report context.

In-situ measurement methods, such as EMA and *μ*EMA, enable participants to annotate their data in real-time, reducing the cognitive biases associated with event recall. Voice-based, open-ended *μ*EMA is particularly well-suited for capturing detailed human activity labels, given the complexity and variability of human behavior. We hypothesized that combining voice- and touch-based inputs could mitigate the contextual bias linked to non-responses in voice-based *μ*EMA, while maintaining a high participant response rate and allowing the collection of a diverse range of postures and activities.

## 3 Multimodal *μ*EMA: System design and implementation

**Figure 1:**
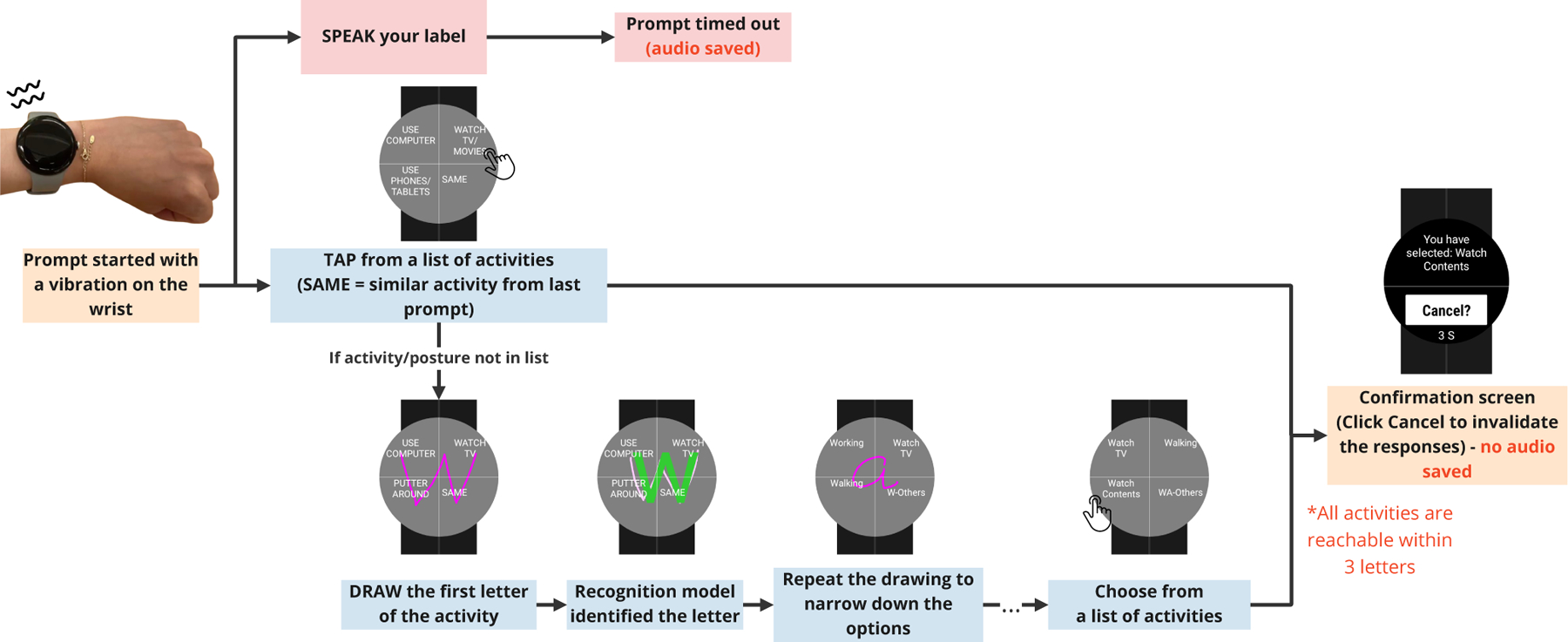
Multimodal *μ*EMA allows users to self-report their posture and physical activity labels using either speech or touch input on a smartwatch. When prompted using watch vibration, participants can complete the survey by either (1) speaking normally (being recorded by the watch) or (2) tapping buttons or drawing letters then tapping a button on the watch to complete the survey.

The goal of the multimodal *μ*EMA system described in this work is to collect temporally-dense activity and posture labels from participants while maintaining high compliance. To achieve this goal, we allow participants to answer the prompt using either touch or speech input (Figure 1). In this section, we outline the components of our system and discuss their implementations.

### 3.1 Prompt design

The smartwatch uses haptic cues to prompt participants to report their in-the-moment posture and activity. The haptic cue is short (~1 s) to avoid distracting participants should they choose to, or need to, ignore the prompt. The cue is ideally intense enough so that participants are unlikely to miss the prompt during bouts of intensive activity or in loud environments. We chose not to use an auditory cue, because sounds from the smartwatch can be overheard by others nearby, potentially causing social disruptions. After the haptic cue, participants have 10 s to start responding to the prompt. To capture temporally dense activity we prompt for *μ*EMA input once every five minutes.

### 3.2 Touch interaction

After the haptic cue, the watch screen displays four quadrants featuring the system’s predictions of the most likely PA or posture labels. The list of four activities shown in the initial screen is determined by the participant’s self-reported most-common activities, a ranked list of which is acquired during a study onboarding session. Subsequently, the system uses the participant’s previous *μ*EMA responses, current heart rate (HR), and the last 10 s of wrist motion (determined using accelerometer data from the watch) to populate the screen. We discuss the details of how we narrow down the options in the implementation section and the appendix of this paper.

If the participant chooses to interact with the system using a touch interaction, they select a physical activity or posture by tapping on the appropriate button quadrant on the watch screen. If the participant chooses to use speech for their previous self-report, then the watch screen will show an option labeled “SAME” in one of the quadrants –this can be pressed to select the last-reported activity/posture again. If the participant uses touch for the prior self-report, then the name of the label that was selected will replace the “SAME” option. If the participant cannot find their in-the-moment activity on the screen, they can draw the first letter (or more) of their activity on the screen over the buttons; this will trigger a search for a different posture or activity that starts with the letter(s). The system then displays the three most probable activities based on the letters drawn. For example, if the participant draws letter “B”/“b,” the screen might show “Biking,” “Bus (Riding),” and “Baking.” If participants draws “CA,” the screen might show “Car (Riding),” “Car (Driving),” and “Carrying Stuff.” Participants can click on the fourth quadrant (“Others”) if they still cannot find the label.

**Figure 2:**
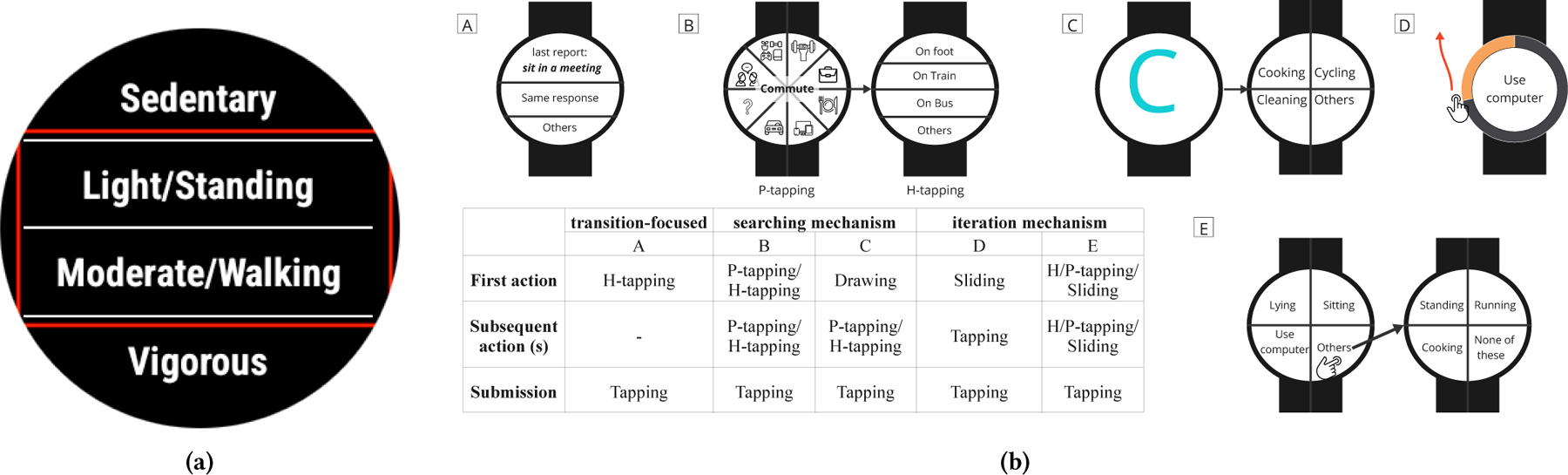
Designing touch interaction. Figure (a) is an example of a *μ*EMA prompt using an h-list; when the buttons are small, the options outlined in red are more difficult to tap than the options at the top and bottom and thus prone to mistakes. Figure (b) shows the different designs proposed in our pilot testing. We considered three interaction mechanism: A) participants tap on “SAME” to indicate if they are doing similar activity with the previous prompt, or “Others” if they cannot find the activity. B and C) participants narrow down the list of labels by tapping on the high-level behavior (e.g., commute, work-related, relaxation) or drawing the first letter of the label. D and E) participants cycled through a list of options by either swiping or tapping on the screen until they found their desired label.

We considered combinations of three different mechanisms and five different interactions on smartwatch self-reporting interfaces (Figure 2b) [113, 114] based on prior work. Our internal pilot testing suggested options A and C were more intuitive and less error-prone than the other input options while still allowing us to include an unlimited number of posture/activity labels. Option A enables users to indicate the same activity/posture to the last prompt, which helps reduce cognitive burden and interaction time. Option C enables users to search for the label by drawing letters on the watch screen. We displayed the list of labels in a radial/pie-list layout (P-list) rather than the traditional horizontal-list layout (H-list) commonly used in previous *μ*EMA studies [85]. This decision was supported by our internal testing, which suggested the H-list layout made the middle options difficult to click due to limited spacing (Figure 2a). In contrast, the P-list layout provides equal space for all four options and maximizes the interaction area.

Participants can cancel their responses if they make a mistake (such as by tapping the wrong option, drawing incorrect letters, or if the system misinterprets the letters). Participants have 10 s to complete the self-report after every letter. Participants can cancel their self-report by selecting the “Cancel?” button at the end of the prompt or at any time by drawing the letter “X” on the watch face. Participants can also simply not answer the prompt.

### 3.3 Speech interaction

In addition to touch interaction, system users can report their postures and activities using speech. We use the same interaction design as prior work on an audio-*μ*EMA system [59]. When the watch prompts the participant, the watch records audio either until the participant reports using touch interaction, or 10 s has elapsed. Because they do not need to even look at the watch or move their hand, participants can maintain their movements while reporting with speech. Although it is not required, even when participants bring their hand closer to their mouth to use the speech input, it is still a one-hand interaction (versus two-hand required for touch).

The system retains audio recordings until participants complete labeling using touch input so that participants can switch to speech input if their desired labels do not appear in the list using touch. When participants opt to report via the touch interaction and complete the interaction, the audio recording for that prompt is deleted to minimize privacy concerns.

### 3.4 Implementation

We implemented the multimodal *μ*EMA system to work on Android Wear devices. For the evaluation study, we loaned participants a Pixel Watch 2 (Alphabet, Inc) and paired the watch to the participant’s personal Android phone, which was running Android 9 or above. The connection to the phone was used to transfer data to our research server during the study. We implemented the system to transfer data from the watch to our server once every hour using Bluetooth and network connections to avoid overflowing the watch storage. From our initial testing, however, the software can function without network connection for up to one month.

We determine the list of activities shown on the watch screen during a touch interaction using previous responses, accelerometer and heart rate (HR) data collected from the smartwatch, and common self-reported activities. The smartwatch estimates physical activity intensity using a real-time algorithm that measures the overall motion of the wrist based on accelerometer data. The smartwatch samples raw tri-axial accelerometer data at 50 Hz and smooths the raw signal using a moving average filter with a window size of 0.5 s (filtered signal). For each axis, it computes the area under the curve (AUC) ${AUC}_{t}=\left|{raw}_{t}-{filtered}_{t}\right|$ to compensate for the effect of gravity (DC offset for the axis) and calculates a 10 s summary of AUC by summing AUC values from the three axes to derive a physical activity summary unit [57, 59]. We implemented the system to use a combination of HR and accelerometer data to account for variability in participants’ heart rate level and wrist motion during sedentary activities (e.g., hand gesturing during conversation). More details about how the system determined the suggested activities are explained in Appendix A.

If participants decide to draw on the watch screen to narrow down the labels, the system uses a predefined activity and posture list to narrow the search. The research team predetermined the mapping between letter sequences and activities/postures. Four research team members independently coded common activity abbreviations; and we used the abbreviations to set the mapping. We derived the list of common activities from the 2024 Adult Compendium of Physical Activities [37] and added in additional activities identified during our internal pilot testing. We included the list of labels in our system in the supplementary materials. We used Google Firebase’s digital ink recognition model ^1^ (Alphabet, Inc.) to identify drawn letters.

## 4 STUDY DESIGN

We conducted a mixed-method, seven-day free-living study with 20 participants to evaluate the multimodal *μ*EMA system. The study took place in three parts: a ≈60-minute in-person introduction/training session, a seven-day free-living period where participants used the multimodal *μ*EMA system to record their behavior, and a ≈60-minute exit interview.

To ensure that the study was adequately powered to detect meaningful differences, we conducted an a priori power analysis using the MRT-SS Calculator^2^ for a micro-randomized control trial for 7 days, assuming 120 decision time points per day (i.e., 10 hours of prompting *μ*EMA, a constant randomization probability of 0.5 at each decision point, and an expected availability of 70%). This response rate assumption is based on the response rate from prior works on *μ*EMA and audio-*μ*EMA [38, 43, 59, 85]). We set the desired power at 80% with a significance level of 0.05. Based on these parameters, the analysis indicated that a minimum of 13 participants would be required to detect the hypothesized proximal effect size. Our targeted recruitment of 20 participants exceeded the required sample size.

We recruited participants using posters placed around an academic campus, social media posts, and campus mailing lists. To be eligible to enroll in the study, participants 1) were at least 18 years old, 2) had no cognitive or hearing impairments, 3) were able to read their phone without reading glasses, 4) used an Android phone, 5) were willing to install an Android application developed by the research team on their phone, and 6) were willing to wear a smartwatch provided by the research team for seven days and answer the prompts on the watch. The study protocol was approved by the IRB at Northeastern University. We compensated participants $75 in Amazon gift cards for the study ($20 for each in-person session, and $5 for each day they wore the watch). We targeted recruitment outside the computer science department and non-STEM students. Besides the Amazon Gift Cards, participants in the study did not receive any other incentives, e.g., class credits.

In later sections of the paper, we use the prefix P with a number to denote participants from the field study. We show the demographic summary of the 20 participants in Table 1.

After obtaining informed consent, we began the training session by collecting demographic information about the participant – age, occupation, and self-reported data about daily habits and physical activity level. A research assistant paired the study smartwatch with the participant’s personal Android phone and installed the study application to ensure the system would work during the free-living period. We showed participants a video demonstrating how to use the multimodal *μ*EMA system (included in the supplemental materials). We asked participants to practice answering the *μ*EMA prompts with a research assistant so they could receive real-time feedback on their responses and ask clarifying questions.

The seven-day, free-living portion of the study began the day after the introductory training session. We instructed participants to wear the provided smartwatch during their waking hours (or until the watch ran out of battery), and report their in-the-moment physical activity and/or posture each time they received a prompt, which was every five minutes. Participants could respond to each prompt using either speech or touch input. The system prompted participants two hours before their sleep time to answer a daily burden survey on their phone. The survey asked them to report any instances in which they removed the watch, their experiences with the system that day, and their expected sleep/wake times for the following day. Additionally, they answered four questions related to perceived burden. The burden questions were: “*I feel comfortable wearing the smartwatch*,” “*I easily responded to the smartwatch prompts*,” “*I responded to the smartwatch prompts quickly*,” and “*The smartwatch is easy to learn how to use*.” These questions were adapted from prior work [59] and used a five-point Likert-scale answer, ranging from “Strongly Disagree” to “Strongly Agree.” Through these questions, we attempted to measure participants’ interaction burden, including their comfort with the watch and the ease of responding to prompts, specifically examining perceived response speed and difficulties in formulating answers. We also asked if the watch or the phone needed to be recharged at any time during the day. To gain additional insights about the effects of the prompting frequency, we collected qualitative feedback from participants during the exit interviews. Once a day, a research assistant sent a text message to the participants to remind them to wear the watch and answer any questions or concerns.

At the end of the seven-day period, participants returned the watch and attended an in-person semi-structured exit interview. During the interview, we asked participants about their experiences using the system including difficulties they may have had while using the system, scenarios when they chose to use speech verses touch input to answer the prompts, and factors influencing their willingness to respond to a prompt. Participants also provided general feedback they had on the system and how to improve multimodal *μ*EMA in future deployments. Overall, 20 participants consented to enroll in the study, and all 20 participants finished the 7-day study (no dropouts).

## 5 Analysis Plan

Our analysis tested these following hypotheses and used thematic analysis to analyze the transcript from the exit interviews.

### 5.1 Hypotheses

We tested four hypotheses to quantitatively evaluate research questions **R1** and **R2**. The hypotheses are motivated by prior works on *μ*EMA and EMA. *H1* and *H2* are related to how ***temporal*** factor (day into study) affects participants’ response behavior. *H3* and *H4* are related to how ***passively-measured*** factors affect participant’s response behavior.

- *H1:* Participants’ response rate to *μ*EMA would decrease over time.
- *H2:* Participants’ perceived burden of multimodal *μ*EMA would decrease over time.
- *H3:* There are associations between passively measured contextual factors and participants’ modality choice.
- *H4:* There are associations between passively measured contextual factors and participants’ response rate.

Prior works have shown that day-into-study has a significant effect on *μ*EMA and EMA response rate. Non-response for EMA tends to be lowest in the beginning of a study and then increase as the study goes on [11, 20, 62, 83, 97]. We also hypothesized that perceived burden would decrease over time as participants acclimated to the smartwatch and responding to *μ*EMA.

To examine the effects of passively-measured factors on participants’ modality choices, we choose seven contextual variables: heart rate, wrist movement, location, phone usage, ambient noises, time of day, and day of week. The passively-measured variables were selected based on prior works, as well as the sensing capability on the smartwatch and the phone. Results from prior works on EMA, *μ*EMA, and multimodal interactions and impact on response rates are summarized in Table 2.

### 5.2 Thematic analysis

We performed inductive coding to assess the usability of multimodal *μ*EMA and identify participants’ perceive source of burden. Two authors carefully read each transcript from our field study and performed open-ended coding using inductive coding [26]. The authors coded the transcript independently and met frequently to reconcile disagreement. The codes were generated and improved iteratively. We merged similar codes/themes and removed codes outside the scope of our research. The interrater agreement (Cohen’s kappa) was *κ* = 0.74.

## 6 RESULTS

We report quantitative and qualitative results from the field study to answer each research question (RQ).

### 6.1 RQ1: Assessing the usability of multimodal *μ*EMA

In the field study, we collected 135 days of data from 20 participants. We lost three days of data for P16 and two days of data for P10 because the participants deleted the app before the exit interviews and the devices had not transmitted data to the research server. Participants responded to 11,320 of the 15,635 delivered prompts (72.4%) – averaging 84 prompts per day. We investigated the usability of the multimodal *μ*EMA system on a smartwatch for seven days by calculating compliance and usability metrics (Table 3). We collected responses on the System Usability Survey (SUS) [12] during the exit interview.

We calculated three prompt response metrics (Table 3): 1) compliance rate refers to the number of prompts that participants interacted with (*𝑝𝑟𝑜𝑚𝑝𝑡𝑠𝐴𝑛𝑠𝑤𝑒𝑟𝑒𝑑*) over the number of prompts scheduled based on participants’ wake/sleep time (*𝑝𝑟𝑜𝑚𝑝𝑡𝑠𝑆𝑐ℎ𝑒𝑑𝑢𝑙𝑒𝑑*); 2) response rate refers to *𝑝𝑟𝑜𝑚𝑝𝑡𝑠𝐴𝑛𝑠𝑤𝑒𝑟𝑒𝑑* over the number of prompts successfully delivered (*𝑝𝑟𝑜𝑚𝑝𝑡𝑠𝐷𝑒𝑙𝑖𝑣𝑒𝑟𝑒𝑑*); and 3) success rate refers to *𝑝𝑟𝑜𝑚𝑝𝑡𝑠𝐶𝑜𝑚pl𝑒𝑡𝑒𝑑* over the number of prompts answered by the participants. *𝑝𝑟𝑜𝑚𝑝𝑡𝑠𝐶𝑜𝑚𝑝𝑙𝑒𝑡𝑒𝑑* are touch responses that participants did not cancel, or speech responses that are inteligible to human annotators. We computed the compliance rate as in prior works on *μ*EMA [43, 59, 83]. The completion rate shows the level of engagement the participant had with the system (the percentage of the questions answered among the questions successfully prompted and delivered) while the compliance rate shows how much data the system captured within a waking day relative to what was anticipated. Compliance is an important metric to consider for future studies to deploy multimodal *μ*EMA to annotate activities and postures for the *entire waking day*. Overall, participants were highly engaged with the system, with the response rate of 72.4% (45.7% or 5,087 of the responses were speech input). The battery limitation of the watch explains the gap in compliance and response rate. The majority of the responses were captured successfully by the system (success rate of 99.8%). Among the *𝑝𝑟𝑜𝑚𝑝𝑡𝑠𝐴𝑛𝑠𝑤𝑒𝑟𝑒𝑑*, 6% of the responses were "SAME" (*𝑛* = 670). The majority (94.8%, 128/135) of the canceled touch inputs were recovered by speech input. Even though there was no upper bound on response time for touch input, the average interaction time was 0.3 s.

**Figure 3:**
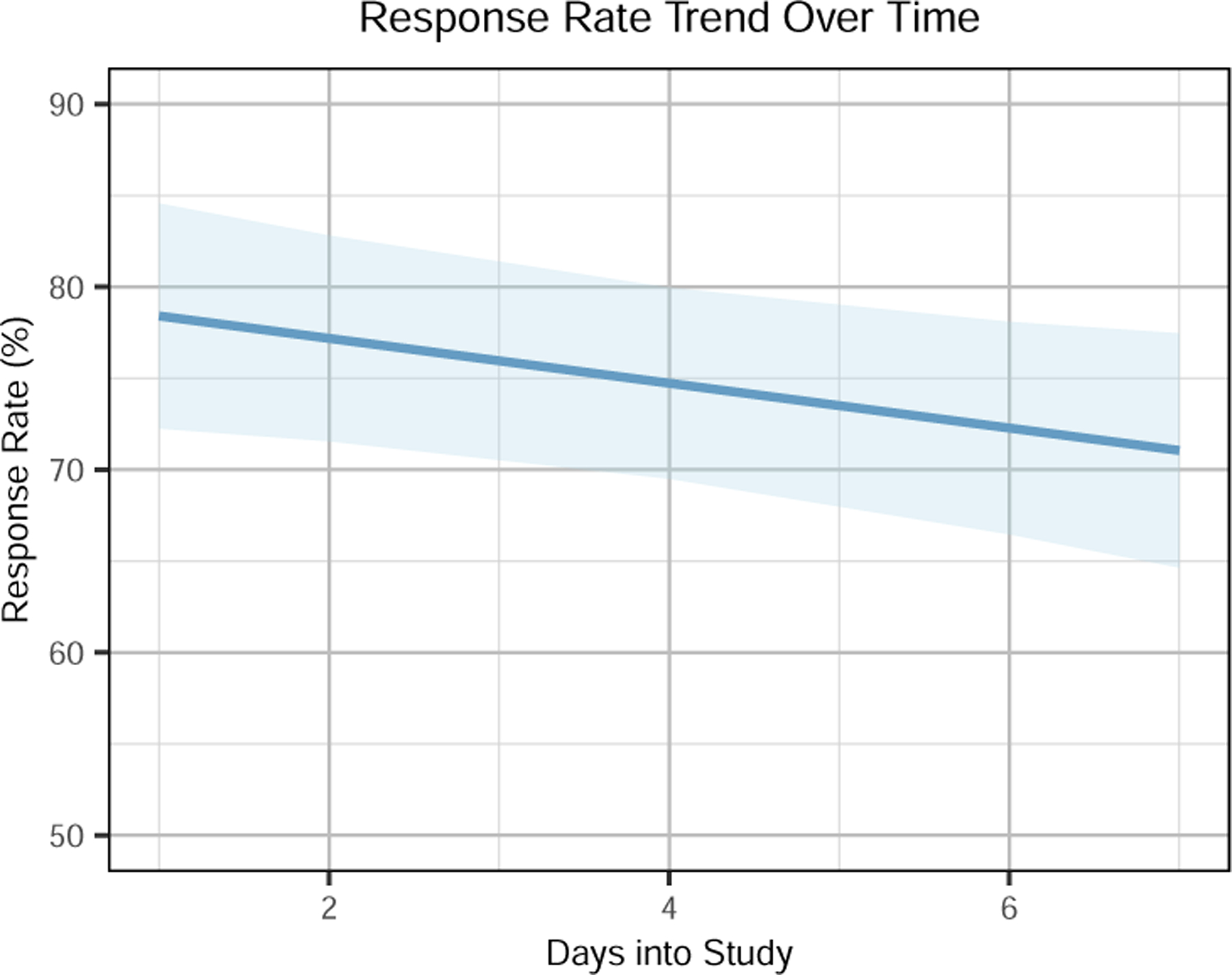
Response rate with regard to day-into-study.

We statistically analyzed whether day-into-study affects response rate over time (*H1)* (Figure 3). We used linear mixed-effect models with random intercept for each participant, with the following formula:

response_rate ∼ day_into_study + (1|subject_id).

Results from the linear mixed-effect model show that response rate decreased over time (*β* = −.01, *𝑆𝐸* = 0.01, *𝑝 <* .05). This indicates that the response rate declined by 1% for each day of the study.

We collected 106 responses for the daily burden survey (response rate: 84.8%). Among the responses, 71 (66.9%) mentioned the watch or the phone needed to be recharged during the day: “*It was quite smooth to use just that the battery [of the watch] would get drained out fast*” [P21]. For each daily burden survey, we converted the Likert responses into a numeric score, with “Strongly Disagree” as 1 and “Strongly Agree” as 5. We statistically tested whether the participants’ responses to individual questions on the daily burden survey changed over time (*H2)*. We fitted linear mixed-effect models for each of the four survey questions with random intercept for each participant, following the formula (*𝑐𝑜𝑛𝑣𝑒𝑟𝑡𝑒𝑑_𝑠𝑐𝑜𝑟𝑒* is the final score converted from the Likert scale for each question):

converted_score ∼ day_into_study + (1|subject_id).

**Figure 4:**
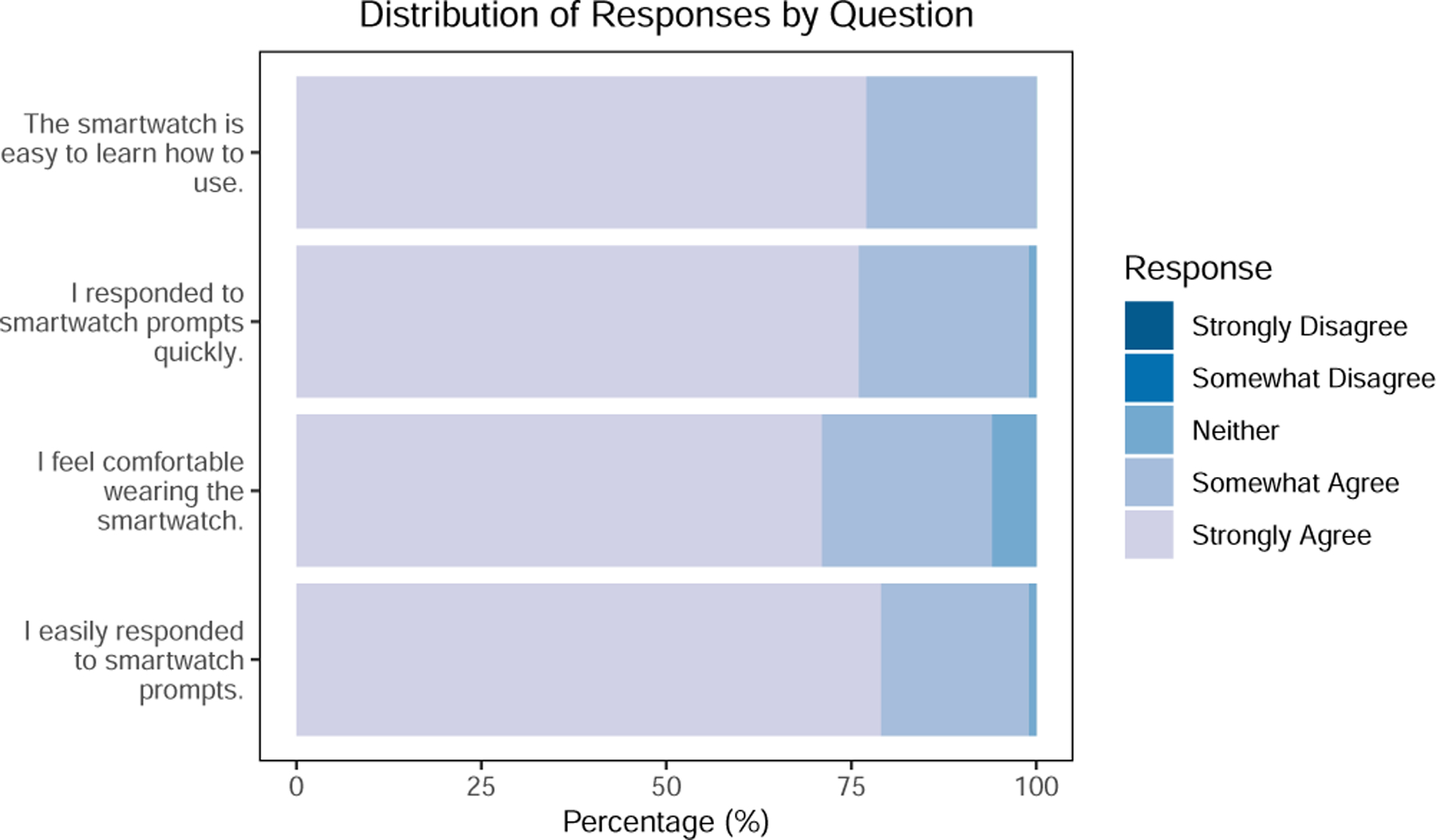
Distribution of responses for individual items in the daily burden survey.

Our results show a decreasing trend over time for question 4: “*I feel comfortable wearing the smartwatch*” (*β* = −.03, *𝑆𝐸* = .02, *𝑝* = .04). This suggests participants feel **less comfortable** with the smartwatch over time, which somewhat contradicts our initial assumption in *H2*. We found no statistically significant result for the other three questions. We show the distribution of responses for each questions in the burden survey in Figure 4.

Our system also received a high usability score (SUS) of 80.1, suggesting high perceived usability [7]. We present the results from the individual questions of SUS in Appendix C.

### 6.2 RQ2: Factors affecting modality choice and response rate in multimodal *μ*EMA prompts

In this section, we quantitatively examine how in-the-moment contextual factors affected participants’ modality choice and response rate *(H3, H4)*. We discuss our qualitative findings on how varying interruption and interaction burden influence participant’s decision making process.

#### 6.2.1 Associations of in-the-moment contextual factors with response rate and modality choice.

**Figure 5:**
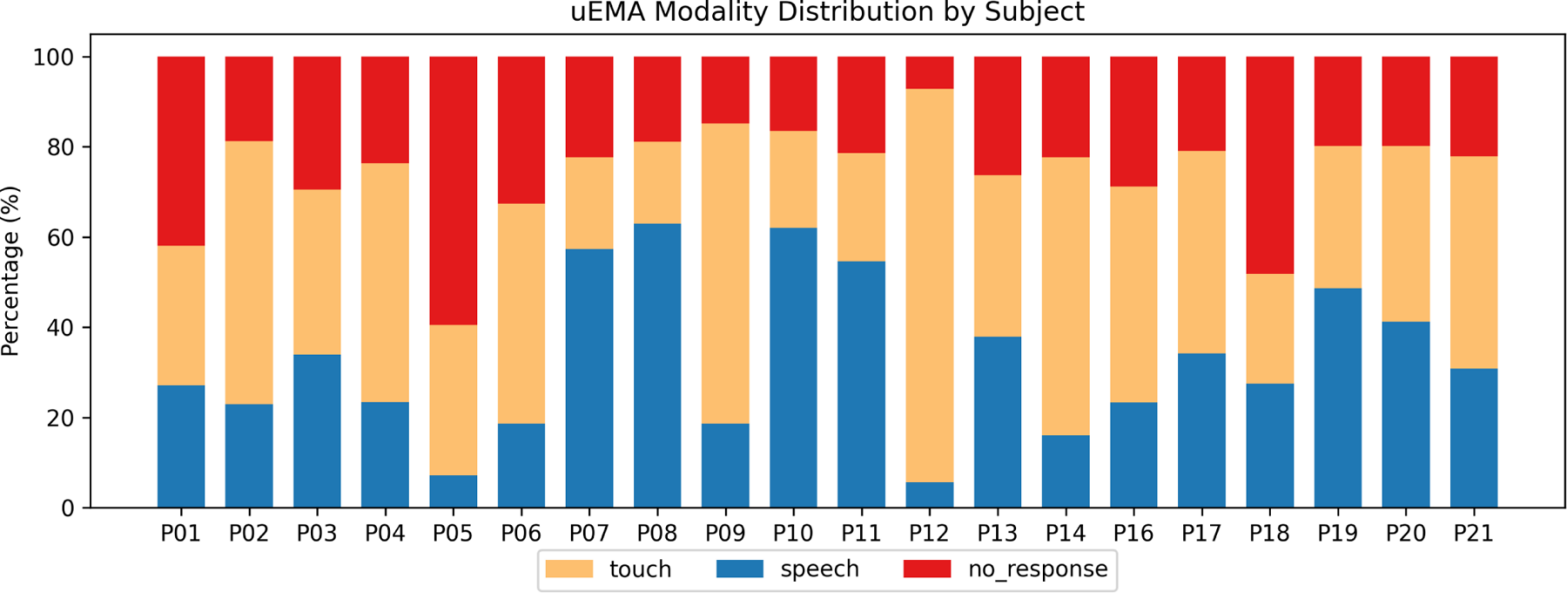
Distribution of modality choice and non-responses for all participants. Even though the ratio of speech to touch input for all participants was relatively balanced (54.7% touch, 45.3% speech), there was large variance between participants. We removed P15 since the participant withdrawn from the study during the consent period.

The distribution of modality usage differs widely across all participants (Fig 5). This variance can be attributed to multiple factors: personal preferences, in-the-moment contextual variables, and prompt interaction/interruption burden.

We used a mixed-effect logistic regression with a random intercept for each participant to predict whether the participant responded to the *μ*EMA prompt (response = 1 vs. non-response = 0). Additionally, we use another mixed-effect logistic regression with a random intercept for each participant to evaluate associations of contexts with modality they opted for (speech = 1 vs. touch = 0). Using the passively collected data, we identified seven in-the-moment contextual variables to use as predictors of non-response and modality choice. These variables include heart rate, wrist movement, location, phone usage, ambiance noises, time of day, and type of day.

##### Heart rate.

We used the heart rate measured by the Pixel Watch 2 at the time of the prompt. If the participants were not wearing the watch at the time of the prompt, we imputed the value with the average heart rate of each participant.

##### Wrist movement.

For wrist movement, we used the AUC unit (as computed in Section 3.4) calculated at the closest time before a prompt. The AUC unit is calculated once every 10 seconds.

##### Location.

The study app recorded GPS data (longitude and latitude) from a participant’s personal phone once every minute. We used the DBSCAN clustering algorithm to identify prominent location clusters where participants spent time during the seven-day study. We labeled the cluster the participants were at most frequently during their self-reported sleep time as “Home,” and other locations were labeled as “Not Home.” We used the location label closest to the time of a prompt as the predictor in our models. “Not home” was the reference variable for the mixed-effect models.

##### Phone usage.

We gathered phone usage data from participants’ personal phones at one-minute intervals. "Phone in use" was set to 1 when a prompt appeared with the phone screen on, 0 otherwise.

##### Ambient noises.

The watch passively listened to 10 seconds of audio before each *μ*EMA prompt and used Google’s YAMNet audio classification model [30] to determine the ambient noises present right before a prompt. The model ran locally on the smartwatch, and no raw audio recordings were saved. We categorized the noises into three mutually-exclusive labels: “Speech,” “Silence,” or “Other noises.” If “Speech” was detected during the 10-second period, the ambient noise of the prompt was set to “Speech.” If “Silence” was the only noise detected by the YAMNet model, the ambient noise label was set to “Silence.” Otherwise, the ambient noise label was set to “Other noises.” “Silence” was the reference variable for the mixed-effect models.

##### Time of day.

We converted the 24-hour time of day into four categories: Morning (6 am to 12 pm), Afternoon (12 pm to 6 pm), Evening (6 pm to 12 am), and Night (12 am to 6 am). “Morning” was the reference variable for the mixed-effect models.

##### Day of week.

We converted each day into “weekday” (Mon-Fri) and “weekend” (Sat/Sun). “Weekend” was the reference variable for the mixed-effect models.

##### Day into study.

Day into study ranged from day 1 to day 7. We show the mixed-effect models below, where *response* is whether the participant responded to a prompt (response vs. non-response), and *modality* is either speech or touch. Results from the mixed-effect models are shown in Table 4.


(1) response ∼ heart_rate + location_ + phone_usage + ambient_noise+time_of_day + day_of_week + day_into_study + (1|subject_id).

(2) modality ∼ heart_rate + location_ + phone_usage + ambient_noise+time_of_day + day_of_week + day_into_study + (1|subject_id).

For modality choice, the main effect of wrist AUC was significant, with *β* = .19 (*𝑆𝐸* = .02, *𝑝 <* .001). This reflects an increase in speech interactions under higher wrist movement. The main effect of heart rate was significant, with *β* = .05 (*𝑆𝐸* = .02, *𝑝* = .05). This reflects an increase in speech interactions under higher heart rate. The effect of location is significant, with *β* = .74 (*𝑆𝐸* = .05, *𝑝 <* .001). This reflects an 75% increase in speech interactions when participants were at home compared to not at home. The effect of ambient noise (detecting “speech” in the environment) was significant, with *β* = −.16 (*𝑆𝐸* = .03, *𝑝 <* .001). This indicates lower speech interactions when detecting speech or conversation noise in the background, compared to silence. The effect of ambient noise (detecting noises other than “speech” in the environment) was significant, with *β* = .01 (*𝑆𝐸* = .03, *𝑝* = .02). This indicates slightly higher chance of speech interactions when detecting noises other than speech or conversation noise in the background, compared to silence. This is because the majority of noises detected in this case are noises coming from activity of the wrist, such as hand washing. Lastly, the effect of day into study was significant, with *β* = −.11 (*𝑆𝐸* = .02, *𝑝 <* .001). This reflects a decrease in speech interactions as the study progresses. We observed no statistical significance for other variables.

For response rate, the main effect of heart rate was significant with *β* = −.26 (*𝑆𝐸* = .09, *𝑝* = .006). This reflects a decrease in response under higher heart rate. The effect of location is significant (“Home”), with *β* = .34 (*𝑆𝐸* = .16, *𝑝* = .03). This reflects an increase in response when participants were at home. The effect of afternoon *β* = −.23 (*𝑆𝐸* = .1, *𝑝* = .05) and evening *β* = −.36 (*𝑆𝐸* = .2, *𝑝* = .001) were significant, indicating that participants were more likely to respond to the prompt in the morning compared to later in the day. We observed no statistical significance for wrist AUC, phone usage, detecting speech or other noises in the background, weekday and during night time.

#### 6.2.2 How contextual variables and modality choice influences interruption and interaction burden of μEMA prompts.

Based on the thematic analysis, we identified two major burdens associated with our system (Table 5): the **interruption burden** and the **interaction burden**. We further identified two different sub-categories associated with the interruption burden (*cognitive* and *social* burden) and three associated with interaction burden (*physical*, *cognitive*, and *social* burden) (Table 5).

##### Interruption burden

Interruption burden refers to the burden produced by the *μ*EMA reporting cue. While the interruption burden could remain the same for both modalities of *μ*EMA, the level of burden depends on the in-the-moment context of the participants. There are two different aspects to the interruption burden. The *cognitive* interruption burden ( cognitive interruption ) occurs when participants receive a prompt during a cognitively engaging activity: (e.g.,“*I am in complete zone like for most of the [computer] games. For an example like when crucial [moments] and the watch vibrate, I actually get irritated.*” [P14]). The *social* interruption occurs when participants are in a public setting or around other people, and the prompts break the flow of the conversation or draw attention in a quiet public space ( disturbing others ).

##### Interaction burden

Interaction burden refers to the burden perceived when participants interact with the *μ*EMA system. The interaction burden can vary based on the participants’ contextual state and modality choice. Participants experienced a *physical* interaction burden when they brought their hand close to their mouth when speaking to the watch, or when they used one hand to tap/draw on the watch face during a touch interaction. Participants commented that the physical interaction burden tended to be higher for touch input when they were moving ( movement/activity ), or when their hands were busy ( hand availability ) (“*since [...] my hands are busy, I won’t tap*” [P1]). Participants’ reactivity to the prompt can cause unnecessary physical interaction burden, which might warp the perceived interaction time of the prompt. Interestingly, we found that participants disagreed over the perceived interaction time between the speech and touch input. Six participants mentioned that speech is noticeably faster (“*I feel it’s [speech] a faster process.*” [P20]). Yet, five participants believed one-tap interaction was faster (“*I click the button I’m standing or walking most of the time, so it’s faster.*” [P14]), since they already have an intuition to look at the watch’s notifications (“*when it [the watch] vibrates* [sic] *, it’s human nature to then see the watch* ” [P14]). The intuition of looking at the watch screen or bringing the watch close to the mouth when a prompt occurs can influence participants’ perception of how long the interaction takes, which could affect modality choice. The *cognitive* interaction burden refers to the mental effort needed to respond to the prompt. Participants raised two major cognitive interaction burdens with *μ*EMA. The first was their mental bias/uncertainty about what to report (“*I do agree that having the options [on the screen] and responding to that lessens the cognitive load because then I’m like, oh, these are my options.*” [P10]) and the quality of their self-report (“*I felt like what I said may not be caught by the system itself [because of] my accent cannot be caught by the system. That could potentially lead to a mislabeling.*”[P12]). The second cognitive interaction burden was the repetition fatigue produced by consecutively giving the same labels over a long period of time. While our daily burden survey did not capture the interruption burden from prompt frequency, our qualitative evaluation from the exit interviews show that 11 (55%) participants reported the repetitive labeling over time as the primary source of burden (not the prompt frequency), seven (35%) expressed a desire for longer intervals between prompts, and two (10%) reported no concerns about prompt frequency. This implies that the repetition fatigue could be related to (or potentially caused by) the intense prompting interval of our study (“*You have to give multiple answers of the same activity [...] For example, you are using your laptop for an hour straight, then once every 5 minutes, you have to give [...] 10 similar prompts*” [P8]). Finally, the *social* interaction cost refers to their social discomfort of the interaction, such as being considered rude to interact with the watch during a conversation or a meeting (“*In public, I feel like when you look at the watch and tap, it’s sort of rude. Like it looks like you’re responding to a message or something.*” [P8]; “*You know close [intimate] conversations, at that time I will tap instead of speak.*” [P1]).

### 6.3 RQ3: Examine the characteristics and potential utility of labels collected using multimodal *μ*EMA

We collected 11,320 labels from the field study. For the speech self-reports, we manually listened to all audio recordings collected from the *μ*EMA prompts and extracted the posture, activity, and context labels from each self-report. We grouped each self-report into one of the five (not mutually exclusive) categories:

- *Singleton posture:* self-report only containing posture label – no activity included
- *Singleton activity:* self-report only containing activity label – no posture included
- *Posture and activity:* self-report containing both posture and activity
- *Multiple activities:* self-report containing multiple activities – may or may not contain posture label
- *Context included:* self-report including contextual information (e.g. location)

We present the distributions of categorized self-reports in Table 6. Because *μ*EMA is limited to a single-question, single-tap response, participants can only report activity *or* posture during touch input, not both. Quantitative results from the field study show that participants tended to choose to report posture over activity in touch responses (of all touch activity labels, 41% of them are “Walking”). From the qualitative findings, we identified three major reasons for this tendency. First, participants might perceive posture labels as more helpful to the researchers ( mental bias/uncertainty ) (“*I think the posture is given first priority more than the activity, right? Because that’s how the audio cues [instructions for the speech input] are. First is the posture then activity.*” [P17]). Second, participants expressed hesitation in searching for activity because there was no guarantee that the label was present in the system ( mental bias/uncertainty ) (“*Regarding writing, the main issue was that I didn’t know what are the exact all categories of things that’s available to me.*” [P14]). Finally, participants reported tapping on whatever options were closest to their activity/posture that appeared on the first screen and could be answered with a simple tap ( reactivity ) (“*When you’re in a hurry, you just look at the screen and then tap on whatever most relevant*” [P16]). These findings show that the interaction cost affects compliance, modality choice, *and* the content of participants’ responses.

To further understand the characteristics of the labels collected from the field study, we manually categorized each label into 10 high-level categories. Additionally, for each self-report, we labeled it as either “macro-label” or “micro-label.” “Macro-labels” are high-level posture and activity labels (e.g., “sitting,” “standing,” “cooking,” “cleaning,” “grooming”). “Micro-labels” are either 1) macro-labels with more context that could potentially influence the sensing signal (e.g., “lounging” (a form of “sitting”), “walking and carrying groceries”) or 2) a micro-activity of a “macro-label” (e.g., “chopping vegetables” vs. “cooking,” “applying lotion” vs. “grooming”). Different HAR datasets/models might focus on detecting or collecting data about macro-labels [56, 93, 115] or micro-labels [1, 2, 15, 100]. Micro-labels can also potentially help debug HAR models, by providing additional contexts that could influence the signal quality (e.g. “standing” vs. “standing and washing dishes”). Table 7 shows the distribution of reported macro-labels and micro-labels.

We categorized the self-reports after completing the data collection period. The categorization process required significant time to parse through and consolidate the list of labels. In HAR data collection studies, however, annotation schemes are likely determined beforehand. After-the-fact label cleaning does not enable real-time feedback or real-time training/monitoring of HAR models. To show the potential for automatic real-time label extraction, we report the results from two tasks: 1) automatic speech recognition (ASR) on the audio recordings collected from the field study, and 2) automatic mapping from users’ self-reports to a pre-determined list of ADL labels used by two publicly available HAR datasets.

For the ASR tasks, we customized a commercial ASR model, Google Cloud speech-to-text v1. We used model adaptation to improve the accuracy of the model and tune the model to recognize targeted word/phrases (e.g., “sitting” is recognized more often than “setting” or “city”)^3^. The watch sent the audio recordings to Firebase and retrieved the transcription on the watch. Due to the unpredictability of network/cellular connections, the ASR process was not guaranteed to be completed in real-time. If there was no network/cellular connection on the watch, the system would stop transcribing to avoid battery drain. To evaluate the usability of ASR, we followed a similar evaluation process as the prior work on audio-*μ*EMA [59]. A human annotator transcribed the audio recording manually to extract the posture/physical activity labels from the recordings. If all posture/activity/context labels were presented in the ASR output, that result was classified as a “correct” ASR transcription. On average, the accuracy of the ASR was 85.9% (*𝑆𝐷* = 5.9 between subjects), significantly higher than the accuracy observed in prior work on audio-*μ*EMA that used off-the-shelf ASR (20–25% accuracy) [59]. Although this can be attributed largely to the changes made to the ASR model, participants’ bias and uncertainty about the audio quality could potentially lead to overall better audio recordings collected (e.g. participants not using speech input in noisy environment, or participants with heavy accents opting to use touch input).

To evaluate the potential for automatic mapping of labels to a target set of labels, we used an open-source large language model (LLM), llama3–8b [105], and prompted it to map the participants’ self-report open-ended labels to two different label lists used by large ADL HAR datasets. By leveraging the LLM’s embedded common sense reasoning about relationship between concepts, we hope it can manage the variability in participants’ self-reports and improve the mapping to structured label sets [80]. CAPTURE-24 is a large scale wrist-worn accelerometer activity dataset collected in-the-wild [14]. Pirsiavash and Ramanan (P&R) is an egocentric camera activity dataset collected in a lab-based setting [81]. The label list used in CAPTURE-24 consists primarily of high-level (macro-labels), while the annotation scheme in the P&R dataset is more detailed and descriptive (micro-labels) ^4^. The P&R dataset, however, only contains home-based activities, while the CAPTURE-24 annotation scheme covers activities outside the home (e.g., “vehicle,” “walking”). If the dataset label list did not contain basic postures (“sitting,” “standing,” “kneeling,” “bending over,” “lying”) or “walking,” we added those labels to the label list. We also added “other” as a category. We include the list of labels in both annotation schemes we used in our experiments in Table 8.

For each self-report collected from the participants, we ran a prompt through llama-3 (see Appendix B) to obtain the mapping of the raw self-report response to the respective annotation scheme. One research team member went through the same process manually and we compared the results to that of the LLM. Another research team member went through the same mapping process on a subset of the labels (400/11,320; 3.5%). The inter-rater reliability rate (Cohen’s kappa) between the two annotators was *κ* = 0.99, which indicates substantial agreement between annotators. We identified three common mistakes made by the LLM:

- *Wrong mappings* refer to obvious mistakes made by the LLM (e.g. “standing” is mapped to a “walking” label).
- *Inferences* refer to instances when the LLM tries to infer the mapping from the self-report (e.g., “doing homework” is mapped to “sitting+using computer” (P&R)).
- *Made-up labels* refer to instances when the LLM mapped the self-report to a non-existant label (e.g., “grooming”).

**Figure 7:**
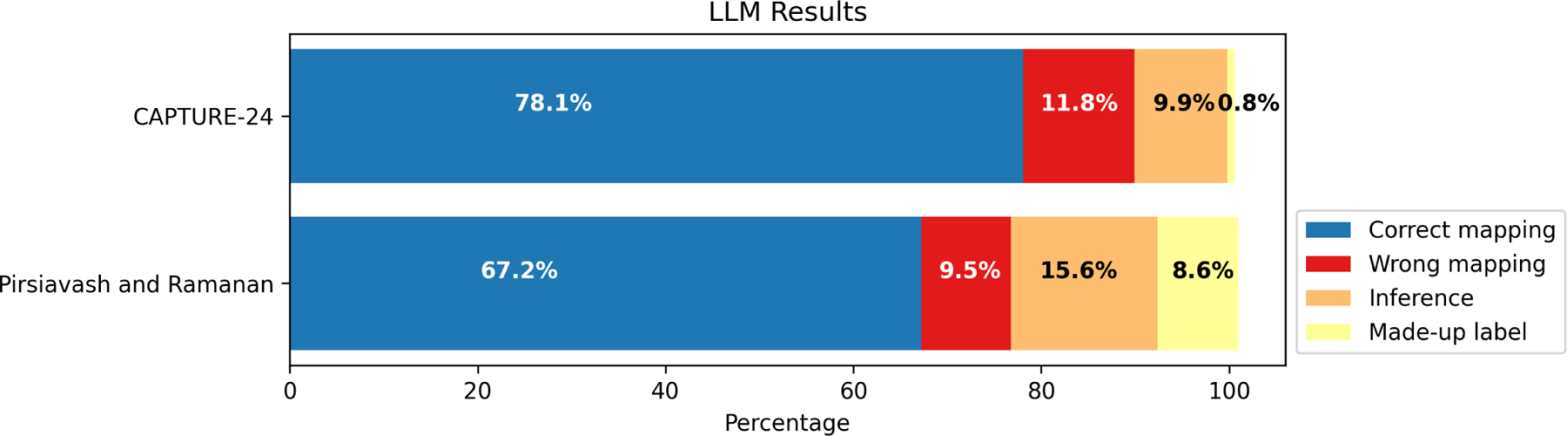
Distribution of correct mappings and mistakes made by the LLM.

Figure 7 shows the distribution of correct mappings and mistakes made by the LLM in the automatic label mapping task. Compared to a human annotator, the LLM has an accuracy of 78.1% and 67.2% for the two annotation schemes. We found that that the distribution of errors between two data set is significantly different (*χ*^2^(3) = 46.5, *𝑝 <* .001). We noticed that the LLM made up significantly more labels using the P&R label list than the CAPTURE-24 list (8.6% vs. 0.8% made-up labels). We believe this was because the P&R label list only contains home activities, so the LLM made up new labels (hallucinations) for self-reports of activities outside the home. For both annotation schemes, the LLM was able to make logical inferences (9.9% and 15.6% inference errors). Even though the inferences might not be always correct in naturalistic settings (e.g., “using computer” might not always associated with “sitting”), these inferences can be useful for future system designs that allow follow-up questions.

## 7 DISCUSSIONS AND FUTURE WORK

We have extended the line of work in *μ*EMA and audio-*μ*EMA by combining two input modalities, touch and speech, into a novel type of *μ*EMA: multimodal-*μ*EMA. In this section, we discuss the implications of this work for future deployments of multimodal-*μ*EMA; design implications for context-aware, multimodal prompting systems; and how our method can be used in a real-time human-in-the-loop activity recognition system.

### 7.1 Usability and Challenges of Deploying Multimodal *μ*EMA in-the-wild (RQ1)

At 15-minute prompting intervals, prior works with touch-only *μ*EMA on a smartwatch reported response rates of 80–90% [43, 85], and speech-only *μ*EMA reported a response rate of 85–90% [38]. Recent work on speech-only *μ*EMA, at a five-minute prompting interval, demonstrated response rates of 65–68% [59]. Despite disrupting participants approximately three times more than standard *μ*EMA, our field deployment showed that participants were able to respond to our proposed multimodal *μ*EMA prompts with a high response rate of 72.4%. Compared to EMA studies of similar duration (7 days) [111], which report an average response rate of 79%, we achieved a comparable level of participant engagement while delivering 10–20 times more prompts (6 vs. 80–120 prompts per day). This shows the promise of using multimodal *μ*EMA implementation on the smartwatch to collect data at high temporal density while maintaining a good response rate.

The majority of participants (55%) expressed it was not the frequency of the prompts, but the repetition of reporting the same labels and the mental load to come up with a response that posed significant cognitive burden [59, 65]. This suggests the possibility of a transition-based prompting mechanism where the system only prompts participants when it detects a change in movement/activity [5, 40, 62, 63, 76]. However, determining the optimal moment to prompt while balancing researchers’ information needs [62] and participants’ burden [71] requires further investigation.

Another major usability challenge we observed during our study was participants’ reactivity to the watch prompt. While in prior work Ruan et al. showed that speech input tends to be faster than touch input [89], our qualitative findings show that participants disagreed about which interaction is faster – speech or touch. Many participants expressed that their ingrained automatic response to a watch prompt was to look at or touch the watch face, even in speech interaction where they were instructed not to. Training people out of this habit is difficult. One possible solution could be to design distinct haptic/auditory cues for the different *μ*EMA prompts. Prior researchers have studied different types of haptic cues and tying them to specific actions/messages [3, 86]. Another option would be to add a new device form factor that *only* has an audio interface and has become quite popular – earables [91]; these devices may prevent participants from looking at the watch screen. Several prior studies have successfully deployed speech-based EMA on earbuds or headsets [9, 59]. Adding additional on-body devices that are not yet socially acceptable in all situations, however, could increase perceived burden by drawing unwanted attention from bystanders.

### 7.2 Disconnection between passive sensing data and perceived source of burden (RQ2)

From the quantitative analysis of our study (*𝐻*4), we observed that higher heart rate, higher wrist movement, and location (at home) have a positive association with the use of speech interaction. Furthermore, presence of speech in the background has a negative association with speech interaction. These findings indicate that people were comfortable using the speech interaction while undergoing high physical activity and movement. Participants were, however, less likely to use the speech interaction if there was background speech (suggesting presence of other people). Our qualitative analyses further support these observations, where participants reported speech input having a lower physical burden, but higher social and cognitive burden compared to touch input. Furthermore, we found that higher heart rate, higher wrist movement, phone usage, weekday, and detecting noise aside from speech in the background are associated with higher *μ*EMA response rate. Participants also are more likely to respond to the prompt in the morning compared to later during the day. These findings are consistent with prior work on contextual biases in *μ*EMA non-response [72, 83].

We want to highlight, however, that there is still a disconnect between the passive sensing data and the interaction burdens raised by our participants [21, 35]. This semantic gap can hinder the deployment of future context-aware system that attempt to predict participants’ receptivity to *μ*EMA prompt [17, 33, 60, 63, 71]. The contextual variables used in our quantitative experiments are often proxies to detect physical and social burden (e.g. location and ambient noises can indicate potential social discomfort , phone usage might indicate indicate hand availability, wrist movement can indicate movement/activity ). These contextual variables, however, do not reveal information about potential cognitive interaction/interruption burden, or all situations where hands were unavailable (e.g., “carrying/moving stuff” or “typing”). Hand availability and repetition fatigue might be detected using fine-grained activity recognition models, and using physiological signals to predict cognitive interruption burden [31]. Burden may exist on a spectrum (e.g., a work meeting likely imposes a higher social burden than a casual conversation with a friend), which passive sensing data may not fully capture. Future systems might automatically measure or estimate burden and explore how it affects participants’ decision-making. Future work should also investigate whether a threshold exists at which burden significantly influences participants’ modality choices.

### 7.3 Towards real-time human-in-the-loop activity recognition systems (RQ3)

Our proposed system, multimodal *μ*EMA, could be useful for real-time annotation and training of activity recognition models [16, 22, 70, 73]. Its flexible input modalities and open-ended responses permit participants to naturally enter what they are doing via speech in a way that allows researchers to gather temporally dense and high-quality labels and subsequently use them to define and tune personalized HAR models. We explored the characteristics of the labels for the behavior labeling task and the possibility of future automation of label extraction. Our findings show that participants were able to report various activity labels under different contexts using our system. We also show that by tuning a commercial ASR and using an open-source LLM we can build a pipeline for automatic label extraction. The LLM performance, however, depends on the list of labels defined by the researchers. Furthermore, participants may have their own mental biases regarding what qualifies as an “activity” or how to search for one using touch input, highlighting the need to co-develop inclusive activity labeling schemes and feedback mechanisms to guide participants in providing useful information (which can also reduce their mental load). In this work, we demonstrate viability of mapping what people report using multimodal *μ*EMA onto desired labels using zero-shot prompting of the LLM. Future work might improve on this method by fine-tuning an LLM model to increase accuracy or by including a re-prompting mechanism to avoid the LLM making up random labels beyond the pre-defined corpus [23].

It is important to note that participants’ mental biases could lead to lower compliance or undesirable reporting patterns for speech interaction (such as overly verbose self-reports or avoiding using speech input), resulting in lower quality of the labels. A real-time feedback loop to combat this bias would depend on the accuracy of the ASR model. Despite the successful use of model adaptation to increase ASR accuracy (86% accuracy), there is still a lot of room for improvement, and the system still requires human supervision to extract all the labels post-hoc. Furthermore, in our current implementation, the ASR model needs a network connection to run, and the network is not always available. We want to emphasize that adding the same confirmation screen used for touch input may not be the optimal solution. Even with a stable network to run the ASR model, the latency of ASR could be too long for the interaction to be considered a “microinteraction.” If the ASR misrecognizes input, it can further increase participant frustration. Instead of using in-the-moment feedback, an end-of-day summary report [42] or making the screen disappear upon detecting the “end-of-speech” could serve as sufficient confirmation mechanism. Future works could further explore the effectiveness of different feedback mechanisms.

## 8 LIMITATIONS

There are some limitations of this work. Many EMA studies only run for a week [20, 97], and thus the seven-day study results reported here provide a baseline for multimodal *μ*EMA use. In this pilot study, a member of the research team checked in daily with participants using text messages. These daily messages built rapport with participants in this short study period, which can increase response rate and compliance with the system [29, 74, 107]. Future studies should assess longer-term, and even longitudinal, use and compliance. The second limitation is that our study populations is skewed towards young male adults who are facile with technology (55% participants self-reported they were very familiar with and regularly used tracking applications on a smartwatch). Our sample size also skewed towards non-Hispanic Asian students, which limits the generalizability of our findings. Future research should study reactions to multimodal *μ*EMA among other groups. A another limitation of this work resulted from hardware restrictions of the Pixel Watch 2; due to the computationally-intensive on-device audio processing and five-minute prompting intervals, the smartwatches running software had a battery life of 12–13 hours before a recharge was required. Future studies could turn off the ambient noise classifier to extend battery life. Furthermore, newer watches continue to have better battery capacity ^5^. Due to a restriction from Google, our watch application required a network connection to run the ASR model; this requirement prevented us from implementing real-time feedback to participants during a speech input. We did not find a significant quantitative association between perceived burden and the day in study (*H2*). This suggests participants might sustain our system over time without increased burden, though our use of unvalidated burden scale may have influenced the results. Future work should look into using other additional measurement for burden/workload, for example the user burden scale or NASA-TLX scale [90, 102]. Our qualitative findings suggest that desire for feedback influenced modality preferences for some participants. A useful future extension to the system might add feedback to the speech responses, either in real-time or via an end-of-day report. Finally, due to the scope of this paper, we did not assess the validity of the labels collected by the system. Given our promising feasibility results, in future work we seek to investigate the usability and validity of the annotations collected using *μ*EMA system, either through an egocentric camera, or using data from the watch sensors [10, 32, 84, 109].

## 9 CONCLUSION

In this paper, we present a novel data collection method, multimodal *μ*EMA, by combining speech and touch input on a smartwatch. We conducted a seven-day free-living study and examined the usability and feasibility of an in-the-wild deployment of our system. Despite the temporal density of the prompts (once every 5 minutes), participants were highly engaged with our system, with an average response rate of 72.4%. We quantitatively and qualitatively identified different factors affecting response rate and modality choice, investigating the characteristics and usefulness of the labels recorded from the field study study. Our field deployment shows the potential of leveraging multimodal *μ*EMA for collecting useful, rich posture and physical activity labels, which can potentially be integrated within a real-time activity recognition system.

## Supplementary Material

List of labels users can search and choose from in multimodal μEMA. This list is only applicable for touch input.

presentation

The tutorial video we showed to the participants on the functionalities of multimodal μEMA.

presentation caption

## Figures and Tables

**Figure 6:**
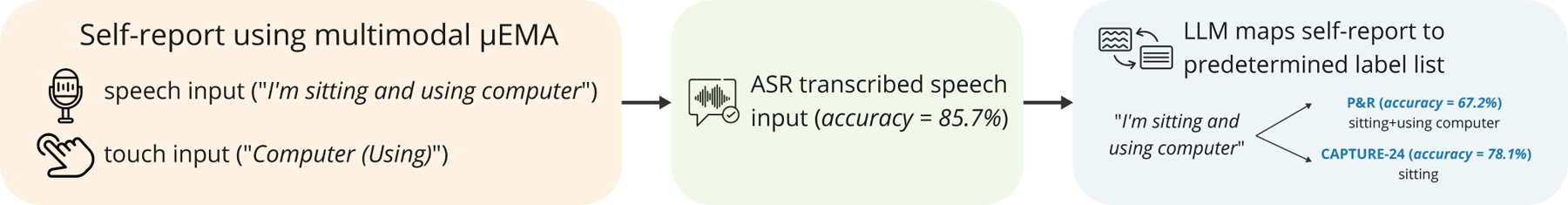
Our proposed pipeline for real-time automatic label extraction.

**Table 1:** Demographics of participants in the field study (N=20). The categories for ethnicity and race are those recommended by the U.S. National Institute of Health [75]. We do not include categories with no participants in the table.

		*N=20*
Age (mean (STD))		25.8 (3.1)
Sex (n (%))	Female	7 (35%)
	Male	13 (65%)
Ethnicity (n (%))	Non-Hispanic	19 (95%)
	Hispanic	1 (5%)
Race (n (%))	Asian	16 (80%)
	White	2 (10%)
	Bi-racial	2 (10%)
Occupation (n (%))	Student	13 (65%)
	Full-time employed	4 (20%)
	Part-time employed	3 (15%)
Daily routine (n (%))	Highly structured	3 (15%)
	Fairly structured	9 (45%)
	Moderately structured	4 (20%)
	Not structured	4 (20%)
Activity level (n (%))	Sedentary	11 (55%)
	Moderate	3 (15%)
	Vigorous	6 (30%)
Familiar with tracking technologies (n (%))	Very Familiar	11 (55%)
	Somewhat familiar	6 (30%)
	Not familiar	3 (15%)
Use a smartwatch (n (%))		7 (35%)

**Table 2:** Summary of prior research examining various passive sensing variables’ effects on response rate and modality choice for EMA and *μ*EMA.

*Contexts*	*Prior Works*	*Passively-Measured Variables*	*Study Findings*
Activity levels	[25, 39, 48, 64,72, 83]	Heart rate, wrist movement	Increased response rates and voice interaction for physical activity group than sedentary group
Phone usage	[83, 108]	Phone interactive	Decreased non-response with recent phone use
Social context	[87, 103, 116]	Detecting speech or conversation in the background	Increased non-response and decreased voice interaction during social interactions
Environmental noise	[39, 72]	Detecting noises in the background	Increased non-response and decreased voice interaction with environmental noise
Location	[88, 99]	Home vs. not home	Decreased non-response at home than other locations
Time of day	[11, 20, 83, 97]	Morning, afternoon, evening, and night	Decreased non-response in the morning than in the afternoon or late evening
Day of the week	[68, 99]	Weekday vs. weekend	Not a statistically significant predictor for response rate

**Table 3:** Usability metrics for multimodal *μ*EMA. #*promptsCompleted* are speech responses that were intelligible to human annotators and touch responses that the participants did not cancel. #*promptsAnswered* are prompts participants interacted with (e.g., an audio input that is not intelligible would be answered but not completed). #*promptsDelivered* are successfully delivered prompts. #*promptsScheduled* are the number of hours between participant’s self-reported wake and sleep times, multiplied by 12. Interaction time measures the duration from the first touch interaction to the last touch interaction. Error recovery rate is the rate of touch responses being canceled and replaced by speech over all canceled touch input.

*Metric*	*Formula*	*All-prompt Mean*	*Between-subject Mean (SD)*
Compliance rate	$\frac{\#promptsAnswered}{\#promptsScheduled}(\%)$	63.1	65.6 (21.4)
Response rate	$\frac{\#promptsAnswered}{\#promptsDelivered}(\%)$	72.4	74.2 (11.5)
Success rate	$\frac{\#promptsCompleted}{\#promptsAnswered}(\%)$	99.8	99.8 (2.34)
SAME rate	$\frac{\#SAME}{\#promptsAnswered}(\%)$	6.01	8.85 (12.9)
SUS scores	—	—	80.1 (11.9)
Error recovery rate	$\frac{\#touchToSpeech}{\#touchCancelled}(\%)$	—	94.8 (1.55)
Interaction time (touch input)	*lastTouch – firstTouch* (s)	0.3 (0.16)	—

**Table 4:** Associations of contextual variables with modality choice and prompt response. We used a mixed-effects logistic regression with random intercept for both experiments. In the touch vs. speech model, we set “touch” to be the reference factor. In the response vs. non-response model, we set “non-response” to be the reference factor. Coefficient converted to an odds ratio (OR) shows how much the odds of an outcome change with a one-unit increase in the predictor, where OR > 1 means higher odds, and OR < 1 means lower odds.

*Variables*		*P*_*speech*_		*P*_*responded*_	
		OR	95% CI	OR	95% CI
Heart rate		1.05 ** 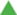	1.0–1.11	0.76 ** 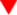	0.63–0.93
Movements	Wrist AUC	1.22 ** 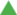	1.15–1.28	1.0	0.85–1.17
Location	Home	2.05 ** 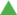	1.84–2.28	1.41 * 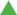	1.01–1.98
Phone usage	Phone in use	1.06	0.95–1.17	1.13	0.8–1.6
Ambience noises	Speech	0.83 ** 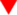	0.78–0.88	0.91	0.76–1.11
	Other noises	1.07 * 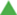	1.01–1.13	1.0	0.83–1.22
Time of day	Afternoon	0.96	0.86–1.07	0.79 ** 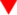	0.53–1.16
	Evening	0.93	0.81–1.05	0.69 ** 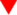	0.45–1.06
	Night	2.04	0.57–7.34	0.03	0-inf
Day of week	Weekday	0.97	0.87–1.08	1.17	0.84–1.62
Day into study		0.89 ** 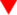	0.84–1.93	0.83 ** 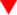	0.74–0.92

** p-value < .001

* p-value < .05.

**Table 5:** Factors influencing participants’ response rate and modality choice. The full definition of the codes are available in Appendix D.

*Type of burden*			*Excerpts from exit interviews*
Interruption	Social	Disturbing others	*[P20] “When there are classes, it could be disturbing during class for others nearby, like sitting next to you, [. . . ] probably the professor would also not be, you know, like willing to have a vibration during class.”*
	Cognitive	Cognitive interruption	*[P21] “I used to try and just mute it in the class because it was just that I wanted to concentrate there. So every five minutes it’s not just productive to [respond] between the lecture.”*
Interaction	Physical	Hand availability	*[P16] “Whenever, let’s say I’m cooking at that time my hands are dirty, or if I’m cleaning or something like that, then my hands are dirty. So a vibration just occurs on my hand and I would speak to the prompt like ’standing cooking.”’*
		Movement/activity	*[P7] “I think that plays into it for sure, like walking the dog or doing dishes or, you know, playing with the dog out in the yard or something like that. If I was up moving around then I would. It’s easier. It’s certainly a lot easier to respond verbally.”*
		Reactivity	*[P10] “Intuitive like having a smartwatch and when you have notifications and you just want to look at the screen or respond or see what the notification is.”*
	Cognitive	Mental bias/uncertainty	*[P2] “Oh, I’m concerned over the data variety basically. So I think from the start if I say like the confusion was like should I say a very detailed approach of like what I’m doing or the type option of sitting or something like that.”*
			*[P12] “When I try to tap something wrong, I was told that if I speak to it before some bits, it might capture the second thing. So I did that, but I don’t know which one it captured exactly. So there’s no confirmation.”*
		Repetition fatigue	*[P8] “Then you just feel a bit silly because you just say the same thing over and over.”*
	Social	Social discomfort	*[P20] “If I’m talking to you, I cannot immediately break your flow or mine [to respond to the prompts]”*

**Table 6:** Distribution of self-reports (% (count)) categorized into five categories based on their contents/labels, sorted by modality. Participants can only report either posture or activity in the touch input.

*Modality*	*Singleton posture*	*Singleton activity*	*Posture and activity*	*Multiple activities*	*Context included*
touch	59.5% (2980)	39.5% (2130)	—	—	—
speech	19.6% (733)	19.4% (723)	46% (1717)	2.2% (84)	6.5% (241)

**Table 7:** The distribution of reported macro-labels and micro-labels.

*Category*	*Macro-labels*	*Micro-labels*
POSTURE (5430)	sitting (3842), standing (1193), bend over (14), kneeling (3), crouching (7), lying (368)	lounging (1), hunching (3), ...
IN TRANSIT (1069)	walking (859), stairs (10), traveling (1), driving (26), ...	going to station (1), riding train (3), going to the station (1), riding electric scooter (1), biking (2), talking/driving (1), waiting for the bus (1), ride plane (1), ...
CHORES (264)	cooking (178), do chores (13), doing laundry (4), cleaning (47), ...	folding stuff (1), carry stuff (2), dusting (1), washing stuff (1), walking/cleaning kitchen (1), putting food away (1), pick up stuff (1), watering plants (2), ...
WORK/SCHOOL (279)	writing (6), doing work (2), meeting (152), working (84), studying (7)	using whiteboard (1), using the whiteboard (1), conversing/writing on whiteboard (1), writing on whiteboard (1), doing an assignment (1), ...
FOOD/DRINK CONSUMPTION (520)	drinking (13), eating (170)	walking/eating (1), eating dinner (2), eating breakfast (2), chopping vegetables (1), drinking water (13), ...
HOBBY (176)	doing crafts (2), shopping (32), reading (35), playing game (31), ...	playing guitar (3), playing frisbee (1), playing video games (15), reading book (18), ...
USE ELECTRONICS (1744)	using computer (94), using phone (85), watch contents (26), use tablet (12), ...	watch a movie (1), working on computer program (1), watch tv (67), use phone/waiting for train (1), typing (50), ...
SELF-CARE (87)	use bathroom (9), grooming (2), ...	wash hands (4), showering (4), washing hands (9), brush teeth (2), taking off socks (1), combing hair (1), wiping face (1), skincare (1), ...
PUTTER AROUND (34)	wake up (1), get ready to walk (1), ready for sleep (2), ...	scratching head (1), locking door (1), climb down ladder (1), charging tablet (2), opening drawer (1), unlocking door (1), ordering food (1), ...
LOCATION (93)	at park (4), around campus (3), to the bus (1), to college (2), in the kitchen (2), around the house (2), in the office (2), outdoors (1), bus connection (1), ...	

**Table 8:** List of labels in datasets used for the automatic self-report to label mapping.

*CAPTURE-24* [14]	*Pirsiavash and Ramanan* [81]
sleep, sitting, standing, lying*, kneeling*, bend over*, household-chores, manual-work, walking, mixed-activity, vehicle, sports, bicycling, others*	sitting*,standing*, lying*, kneeling*, bend-over*, walking*, combing hair, make up, brushing teeth, dental floss, washing hands/face, drying hands/face’, enter/leave room, adjusting thermostat, laundry, washing dishes, moving dishes, making tea, making coffee, drinking water/bottle, drinking water/tap, making hot food, making cold food/snack, eating food/snack, mopping in kitchen, vacuuming, taking pills, watching tv, using computer, using cell, making bed, cleaning house, reading book, using mouth wash, writing, putting on shoes/socks, drinking coffee/tea, grabbing water from tap, other*

* Labels with an asterisk (*) were added by our research team.

## CCS Concepts

- Human-centered computing → Empirical studies in ubiquitous and mobile computing.

## Footnotes

1
https://developers.google.com/ml-kit/vision/digital-ink-recognition

2
https://d3center.shinyapps.io/mrt-ss-calculator/

3
https://cloud.google.com/speech-to-text/ondevice/docs/model_adaptation

4 In this experiment, we used the mapped labels that the authors of CAPTURE-24 used when training/evaluating HAR models [14].

5
https://blog.google/products/pixel/google-pixel-watch-3/

## Appendix

### A Appendix A: Activity level estimation

To decide which activities to display on the screen, we use a rule-based, heuristics algorithm. We assigned each activity in the list into one level: *Sedentary, Moderate and Vigorous*. The categorization and the list of activities are included in the supplementary materials. We determined participant’s activity level using heart rate and accelerometer data before a prompt popped up (see Table 9). We filtered the list of activities that fell into the detected physical activity level, and we displayed the three activities that were ***most reported*** by the participants. If there were ties, we displayed the activities based on alphabetical order.

**Table 9:** How we determined physical activity levels using heart rate (HR) and wrist accelerometer data (AUC unit).

*Activity Level*	*Heart rate (HR) and AUC values*
Vigorous	HR > 150 OR AUC >= 6000
Moderate	NOT Vigorous
	AND (HR >= 105 OR AUC >= 2000)
Sedentary	HR < 105 AND AUC < 2000

### B Appendix B: Prompts used for LLM experiment

To run the LLM experiment, we used the following prompt, which is tailored for the CAPTURE-24 label list. To run the experiment with the P&R label list, we simply replaced the list of labels (see the *italicized text*).

This is what the person reported doing: [participants’ self-report].

Map their self-report to one or more of the following labels:

sleep,

sitting,

standing,

lying,

kneeling,

bend over,

household-chores,

manual-work,

walking,

mixed-activity,

vehicle,

sports,

bicycling,

others.

If the self-report fits multiple labels, seperate them with a backslash (such as sitting/household-chores, standing/sports, sitting/vehicle, ...). If the self-report doesn’t fit any of the labels, return ’others’. Give a single answer (no explanation, limited prose). Do not invent new labels.

The prompt for the P&R experiment is identical, except for the activity list is replace with these activities: sitting,standing, lying, kneeling, bend-over, walking, combing hair, make up, brushing teeth, dental floss, washing hands/face, drying hands/face’, enter/leave room, adjusting thermostat, laundry, washing dishes, moving dishes, making tea, making coffee, drinking water/bottle, drinking water/tap, making hot food, making cold food/snack, eating food/snack, mopping in kitchen, vacuuming, taking pills, watching tv, using computer, using cell, making bed, cleaning house, reading book, using mouth wash, writing, putting on shoes/socks, drinking coffee/tea, grabbing water from tap, other

### C Appendix C: Results of individual items in the SUS survey

Table 10 shows the results of individual SUS items.

### D Appendix D: Themes, Codes and definitions (qualitative analysis for participant’s perceived burden)

Table 11 shows the definitions of the themes mentioned in the qualitative analysis (Section 6.2.2), as well as exampled generative codes.

**Table 10:** Individual SUS questions with mean and standard deviation of the score. The scale for grading is from 0–4. The table shows the raw scores recorded in the SUS response (“Strongly Agree” is graded as 4, and “Strongly Disagree” is graded as 0).

*Question*	*Mean*	*SD*
I think that I would like to use this system frequently.	2.73	1.10
I found the system unnecessarily complex*	1.2	0.94
I thought the system was easy to use.	3.27	0.80
I think that I would need the support of a technical person to be able to use this system.*	0.47	0.74
I found the various functions in this system were well integrated.	3.00	1.00
I thought there was too much inconsistency in this system.*	1.13	0.92
I would imagine that most people would learn to use this system very quickly.	3.33	0.82
I found the system very cumbersome to use.*	1.07	0.88
I felt very confident using the system.	3.27	0.70
I needed to learn a lot of things before I could get going with this system.*	0.6	0.74

* For the even numbered questions (marked with * in the table), the raw scores are reverse graded (by subtracting the score from 4) – so lower score on these question implies high usability. The final SUS score is calculated by summing up all the grade for individual questions, and multiplying by 2.5.

**Table 11:** Definitions of the codes about participant’s perceived source of burden emerged from the qualitative analysis.

*Type of burden*			*Example codes (count)*	*Definition*
Interruption	Social	Disturbing others	high perceived social disruption (10)	Situations where the prompting cue causes disruptions for people around the participants
	Cognitive	Cognitive interruption	prompt during activity transition is cognitively burdensome (3), annoyed with prompts during focus time (5)	Situations where participants claimed the prompts interrupted them from a cognitively engaging task
Interaction	Physical	Hand availability	speech preferred when hands are busy/moving around (14)	Situations where participants’ hand movement affected their modality choice or non-response
		Movement/activity	speech was helpful to avoid looking at watch screen during activity (2)	Situations where participants’ activity level affected their modality choice or non-response
		Reactivity	instinct to look at screen during watch prompt (7), tendency to bring watch hand near mouth for speech (2)	Situations where participants’ reaction to the prompt affected their modality choice or non-response (e.g., a change in body movement, bringing their hand close to their mouth, looking/glancing at the watch)
	Cognitive	Mental bias/uncertainty	hard to come up with response for speech (6), audio quality concerns (20)	Situations where participants expressed doubt/uncertainty about what to report or the quality of the recorded label
		Repetition fatigue	repetition is burdensome (11), increase time between prompts (7)	Situations where participants complained about the repetition of the same label
	Social	Social discomfort	study participation details not shared in professional settings (2), non speech inputs preferred around others (5)	Situations where answering to the prompting caused participants to be uncomfortable around other people
