# Supplementary material for: Feasibility and Utility of Multimodal Micro Ecological Momentary Assessment on a Smartwatch
Source: Proc SIGCHI Conf Hum Factor Comput Syst. Author manuscript; Available in PMC 2025 Dec 22. (PMC12718675; doi:10.1145/3706598.3714086)
Supplement: List of labels users can search and choose from in multimodal μEMA. This list is only applicable for touch input. [file NIHMS2119385-supplement-List_of_labels_users_can_search_and_choose_from_in_multimodal__EMA__This_list_is_only_applicable_for_touch_input_.pdf]

Activity categories	Variation shown on the watch	Variation shown on the watch	Letter that goes with the variation (for searching)
Posture	Sitting	Sitting	S
	Lying	Lying	L
	Standing	Standing	S
		Upright	U
	Kneeling	Kneeling	K
	Crouching	Crouching	C
	Jumping	Jumping	J
	Bending Over	Bending Over	B
Commute/Travel	Airplane (riding)	Airplane (riding)	A
		Plane (riding)	P
	Scooter (riding)	Scooter (riding)	S
	Skateboard (riding)	Skateboard (riding)	S
	Bike (riding)	Bike (riding)	B
	Electric Bike (riding)	Electric Bike (riding)	E
	Bus (riding)	Bus (riding)	B
	Train (riding)	Train (riding)	T
	Subway (riding)	Subway (riding)	S
	Motorcycle (riding)	Motorcycle (riding)	M
	Motorcycle (driving)	Motorcycle (driving)	M or D
	Riding car	Car (riding)	C
	Driving car	Car (driving)	C or D
	Riding boat	Boat (riding)	B

	Riding hoverboard	Hoverboard (riding)	H
	Riding unicycle	Unicycle (riding)	U
	Riding elevator	Elevator (riding)	E for Elevator
	Riding escalator	Escalator (riding)	E for Escalator
Exercise	Aerobics	Aerobics	A
	Crossfit	Crossfit	C
	Cycling	Cycling (outdoor)	C
		Cycling (indoors)	C
	Yoga	Yoga	Y for Yoga
		Aerial Yoga	A for aerial, Y for Yoga
	Weightlifting	Weightlifting	W
		Lifting Weights	L
		Calisthenics	C
	Strength training	Strength training	S
	Zumba	Zumba	Z
	Barre	Barre	B
	Pilates	Pilates	P
	Use treadmill	Treadmill (On)	T
	Use Stairmaster	Stairmaster (On)	S
		Stationary Bike (On)	S or B
	Use elliptical	Elliptical (On)	E
	Use rowing machine	Rowing Machine (On)	R
	Erg (On)	Erg (On)	E

		Jumping Rope	J
	Stretching	Stretching	S
	Running	Jogging	J
		Running	R
		Bodyweight Workout	B
		Resistance Training	R
	Hiking	Hiking	H for Hiking
	Kickboxing	Kickboxing	K
	HIIT	HIIT	H
	Boxing	Boxing	B
Use bathroom	Use Bathroom	Use Bathroom	B for bathroom
Grooming	Brushing teeth	Brushing teeth	B for brushing, T for Teeth
	Flossing	Flossing	F
	Doing Nails	Doing Nails	N
	Shaving	Shaving	S
	Applying Makeup	Applying Makeup	M
	Waxing	Waxing	W
	Removing Makeup	Removing Makeup	M or R
	Massaging	Massaging	M
	Getting Massage	Getting Massage	M for massage
	Washing Hands	Washing Hands	W for Washing, H
	Washing Face	Washing Face	W, F
	Styling hair	Styling hair	S for styling/H for Hair
	Drying hair	Drying hair	D for drying, H for hair
	Applying	Applying Skincare	S

	Skincare		
Taking Medication	Taking Medication	Taking Medication	M
Changing Clothes	Changing Clothes	Changing Clothes	C
		Getting Dressed	D
Laundry	Folding Clothes	Folding Clothes	F or C
		Putting Away Clothes	C
	Doing laundry	Loading Machine	W for washing machine?
		Unloading Machine	W for washing machine?
	Handwashing Garment	Handwashing Garment	H, W
	Ironing	Ironing	I
	Steaming	Steaming	S
	Hanging Clothes	Hanging Clothes	C or H
	Lint rolling	Lint rolling	L
	Shining Shoes	Shining Shoes	S
Eating	Eating	Eating	E
		Eating Breakfast	B for breakfast
		Eating lunch	L for Lunch
		Eating Dinner	D for Dinner
		Snacking	S for Snack
Walking	Walking	Walking	W
		Brisk walking	B
		Slow walking	S
Kitchen Activities	Cooking	Cooking	C
	Putting away food	Putting away food	F
	Chopping Food	Chopping Food	C

	Baking	Baking	B
	Washing Dishes	Washing Dishes	W or D
	Loading Dishwasher	Loading Dishwasher	D
	Unloading Dishwasher	Unloading Dishwasher	D
	Grilling	Grilling	G
Clean	Cleaning	Cleaning	C for cleaning
		Dusting	D for dusting
		Mopping	M for mopping
		Sweeping	S for sweeping
		Wiping Surface	W
		Vacuuming	V
		Organizing Cabinet/Shelf	O or C
		Scrubbing Surface	S
Computer/Screen	Using Computer	Using Computer	C
	Using Phone	Using Phone	P
	Using Tablet	Using Tablet	T
	Typing	Typing	T
	Play Video Game	Play Video Game	P for Playing, V for Video
	Phone Call	Phone Call	P or C
	Watch Content	Watch Content	W
		Watch Movie	M
		Watch TV	T
		Watch Video	V
Leisure Activities	Reading	Reading	R
	Spikeball	Spikeball	S

	Playing Catch	Playing Catch	P, C
	(Doing) Puzzle	(Doing) Puzzle	P
	Meditating	Meditating	M
Talking	Talking	Talking	T
		Conversing	C for conversing
		Speaking	S
		Chatting	C for chatting
Writing	Writing (by hand)	Writing (by hand)	W or H
Pet-related	Play with pet	Play with pet	P for (Play) and P (for Pet)
		Play with Dog	D (for Dog)
		Play with Cat	C (for Cat)
	Walking Dog	Walking Dog	D for Dog
	Grooming Pet	Grooming Pet	G
	Washing Pet	Washing Pet	W
Stairs	Stairs (Up)	Stairs (Up)	S for stairs, C for climbing
	Stairs (Down)	Stairs (Down)	S for Stairs
Stuff	Carrying Stuff	Carrying Stuff	C for carrying
		Moving Stuff	M for moving
	Pack Stuff	Pack Stuff	P for pack
	Unpack stuff	Unpack stuff	U for unpack
	Lifting Stuff	Lifting Stuff	L
	Hanging Stuff	Hanging Stuff	H
Misc Chores	Watering Plants	Watering Plants	W for water
	Mowing Lawn	Mowing Lawn	M
Playing Game	Playing Game	Playing Game	P for Playing, G for Game
		Board Gaming	B for board, G for game

Shopping	Shopping	Shopping	S for Shopping
		Grocery Shopping	G for Grocery
Meeting	(in) Meeting	(in) Meeting	M for meeting
Sports	Sports	Archery	A
		Bowling	B
		Frisbee	F
		Basketball	B
		Ice Skating	I or S?
		Roller Skating	R or S?
		Figure Skating	F or S?
		Roller Derby	R or D?
		Tennis	T
		Pickleball	P for Pickleball
		Squash	S
		Badminton	B
		Racquetball	R
		Kickball	K
		Martial Arts	M
		Pingpong	P
		Table Tennis	TA for table, TE for tennis
		Volleyball	V
		Netball	N
		Golfing	G
		Gymnastics	G
		cheerleading	C
		Football	F
		Fencing	F

		Soccer	S
		Hockey	H for Hockey
		Baseball	B
		Dodgeball	D
		Softball	S
		Rock Climbing	R for Rock, C for Climbing
		Bouldering	B
		Dancing	D
		Lacrosse	L
		Cricket	C
Outdoor Water Things	Outdoor Water Things	Kayaking	K
		Jet Skiing	J
		Windsurfing	W
		Sailing	S
		Rowing	R
		Fishing	F
		Paddleboarding	P
		Surfing	S
Arts	Painting	Painting	P
	Drawing	Drawing	D
	Sculpting	Sculpting	S
	(Playing) Music	(Playing) Music	M
	(Playing) Instrument	(Playing) Instrument	I
	Doing Pottery	Doing Pottery	P
		Doing Ceramics	C
	Knitting	Knitting	K

	Crocheting	Crocheting	C
	Sewing	Sewing	S
	Quilting	Quilting	Q
Religious activities	Praying	Praying	P
Puttering Around	Puttering Around	Puttering Around	P
Home repair	Hammering	Hammering	H
	Nailing	Nailing	N
	Woodworking	Woodworking	
	Painting (Wall)	Painting (Wall)	P for painting, W for Wall
Fixing	Fixing Stuff	Fixing Stuff	F

Up for debate “activities”:

Listening	Listen to Lecture	L (for lecture)	SED/MOD	LOW LEVEL
	Listen to Music	L for listening, M for music	SED/MOD/ VIG	LOW LEVEL

Note: activities where watch is submerged in water have been excluded (e.g., swimming, diving)
